# A public health framework for reducing stigma: the example of weight stigma

**DOI:** 10.1007/s11673-022-10199-3

**Published:** 2022-07-20

**Authors:** Alison Harwood, Drew Carter, Jaklin Eliott

**Affiliations:** 1 The Office of Research Ethics, Compliance and Integrity, The University of Adelaide, Adelaide, South Australia 5005 Australia; 2 Adelaide Health Technology Assessment, School of Public Health, The University of Adelaide, Adelaide, South Australia 5005 Australia; 3 Associate Professor, School of Public Health, The University of Adelaide, Adelaide, South Australia 5005 Australia

**Keywords:** Stigma, Stigmatization, Obesity, Overweight, Weight stigma, Health behaviour, Obesity prevention and control, Obesity psychology, Prejudice, Public health, Social justice and psychology, Stereotyping

## Abstract

We examine stigma and how it operates, then develop a novel framework to classify the range of positions that are conceptually possible regarding how stigma ought to be handled from a public health perspective. In the case of weight stigma, the possible positions range from encouraging the intentional use of weight stigma as an obesity prevention and reduction strategy to arguing not only that this is harmful but that weight stigma, independent of obesity, needs to be actively challenged and reduced. Using weight stigma as an illustrative example, we draw on prior theoretical work on stigma mechanisms and intervention strategies to develop a framework for improving the understanding, evaluation, and planning of anti-stigma interventions. This framework has the potential to help public health actors to map out how protest, contact, education, and regulation strategies can be used to reduce direct discrimination, structural discrimination, and internalized stigma (self-stigma).

## Introduction

The stigmatization of diseases, conditions, and characteristics has a long history within public health. Tuberculosis, leprosy, HIV/AIDS, cancer, mental illness, and smoking are just some that have been stigmatized (Bayer 2008; Bell et al. 2010; Evans-Polce et al. 2015; Mahajan et al. 2008). Research on these and related stigma has found that stigma acts as a significant and dangerous barrier to seeking or accessing healthcare and itself has harmful effects on physical and mental health (Brown, Macintyre, and Trujillo 2003; Mahajan et al. 2008; Puhl 2011; Puhl and Brownell 2001). Similarly, a growing body of literature has examined weight stigma specifically and documented a range of harmful effects on health, independent of weight (Bertakis and Azari 2005; Brewis 2014; Hatzenbuehler et al. 2013; MacLean et al. 2009; Stuber, Meyer, and Link 2008).

Obesity is a deeply stigmatized condition. Individuals classified as overweight or obese are stereotyped as lazy, undisciplined, incompetent, weak-willed, and gluttonous (Brownell et al. 2010; Puhl, Andreyeva, and Brownell 2008a; Puhl and Heuer 2009). Beliefs that self-indulgence, gluttony, and laziness cause obesity function to hold individuals classified as overweight responsible for their condition (Dejong 1980, 77). As Cahnman reflects, “Clearly, in our kind of society … being overweight is considered to be detrimental to health, a blemish to appearance, and a social disgrace” (1968, 283). Academic literature attests that weight stigma can result in psychosocial harms, including social isolation and discrimination. In turn, these harms can negatively impact a person’s self-esteem, academic achievement, employment opportunities, and health. Many individuals who are perceived to be overweight or obese experience discrimination in interpersonal and structural forms, including social ostracism, disrespectful treatment, and fewer opportunities, owing to differential treatment in areas of employment, education, and healthcare (Hatzenbuehler, Phelan, and Link 2013; Link 2001; MacLean et al. 2009; Musher-Eizenman et al. 2004).

Many academics recognize that intentional stigmatization is both ineffective and morally problematic as a policy option to reduce obesity (Puhl 2011; Salvy et al. 2011; Zabinski et al. 2003). But seldom have governments or other actors actively attempted to reduce weight stigma, and the few attempts that have been made have had mixed results (Bell and Morgan 2000; Haines, Neumark-Sztainer, Eisenberg, and Hannan 2006a; Irving 2000; Musher-Eizenman et al. 2004; Sigelman 1991; Sigelman et al. 1986). In this paper, we develop a conceptual framework to promote the success of anti-stigma efforts.

First, we examine stigma in general and how it operates. We then develop a novel Spectrum of Approaches to Stigma, classifying the range of positions that are conceptually possible to adopt regarding how stigma ought to be handled. In the case of weight stigma, the positions range from encouraging the intentional use of weight stigma as an obesity reduction strategy to arguing both that this is a harmful approach and that weight stigma needs to be actively reduced independently of obesity. We then develop our novel Matrix of Anti-Stigma Interventions, a conceptual framework that stands to improve the understanding, evaluation, and planning of anti-stigma interventions in the case of weight stigma and beyond. Finally, we demonstrate how the matrix can be used to understand past interventions aimed at reducing weight stigma and we highlight promising elements of those interventions. Overall, our method is to mount an ethical argument and to make theoretical advancements by drawing on prior theories and empirical studies. Our argument is that weight stigma ought to be intentionally reduced, and the spectrum and matrix that we innovate are frameworks for understanding how public health actors can, respectively, handle and combat stigma, including weight stigma.

## Stigma and How it Operates

There is variation in how stigma is defined, in part due to two things. First, the concept of stigma has been applied to a wide variety of things, such as mental illness, HIV/AIDS, leprosy, disability, cancer, and non-health related issues, such as exotic dancing, IQ, choice of profession, and sexual orientation (Bayer 2008; Bell et al. 2010; Lewis, 1998; Mahajan et al. 2008). Second, a wide variety of analytical tools have been used to examine stigma and its effects, reflecting the multidisciplinary nature of stigma research (Brown, Macintyre, and Trujillo 2003; Klepp et al. 1997; Pinfold et al. 2014).

In his seminal work, Erving Goffman described a stigmatized attribute as “an attribute that is deeply discrediting”; the stigmatized attribute reduces the bearer “from a whole and usual person to a tainted, discounted one” (Goffman 1963, 3). More recently, Link and Phelan have argued that stigmatization is a product of “the co-occurrence of certain interrelated components,” positing that relationships between particular components result in the stigmatization of individuals and subpopulations (2014, 367). These components include distinguishing and labelling human differences, associating those differences with negative attributes and stereotypes, separating “us” and “them,” and the status loss and discrimination experienced by the stigmatized (367–376). Link and Phelan go on to contend that the stigmatization of individuals and sub-populations relies upon “access to social, economic, and political power that allows the identification of differentness, the construction of stereotypes, the separation of labelled persons into distinct categories, and the full execution of disapproval, rejection, exclusion, and discrimination” (375–376). In other words, the dominating values and opinions of one group are expressed in ways that result in individuals who belong to another group being discriminated against.

Link (2001) presents three mechanisms through which stigmatization can have negative consequences for stigmatized individuals: direct discrimination, structural discrimination, and social psychological processes operating through the stigmatized person.

Direct discrimination involves attitudes and beliefs directly issuing in discriminatory behaviour: person A’s stigmatization of something attributed to person B causes person A to engage in overt forms of discrimination against person B (e.g., rejecting their job application or excluding them socially). Structural discrimination refers to inequalities in life chances, not necessarily overt discrimination. Finally, social psychological processes operating through the stigmatized person are also described in other literature in terms of “self-stigma” or “self-stigmatisation” (Barlösius and Philipps 2015; Evans-Polce et al. 2015; Rüsch, Angermeyer, and Corrigan 2005). People develop conceptions of a stigmatized condition such as mental illness as part of being socialized into their culture; these conceptions then become “lay theory.” Expectations are formed as to whether most people will devalue a person with a mental illness and reject them as a friend, spouse, or employee (Link 2001, 10). If a person then goes on to develop a mental illness, they may fear that those expectations will be applied to them (Nolan and Eshleman 2016). When stigmatizing messages become part of an individual’s own outlook, this can have serious negative consequences. For example, fear of rejection may mean acting less confidently, withdrawing from or avoiding particular situations, and having strained and uncomfortable social interactions. In turn, this may cause social networks to be constrained, leading to social isolation, compromised quality of life, unemployment, and income loss. It is important to note that Link’s three mechanisms are mutually reinforcing. For example, as a result of receiving poor treatment via direct discrimination (mechanism 1), an individual may come to expect further poor treatment (mechanism 3).

We use the term “weight stigma” to refer to the stigmatization of individuals perceived to be overweight or obese. A range of terms are used to draw attention to this and related phenomena. These terms include the “stigmatisation of obesity” (Couch et al. 2016), “weight bias” (Browne 2012; Puhl and Brownell 2003; Puhl et al., 2008a, b; Schwartz et al. 2003; Washington 2011), “fat shaming” (Farrell 2011), “anti-fat attitudes” (Hague and White 2005; Puhl et al., 2008a, b), “weight stigma” (Nolan and Eshleman 2016; Puhl and Heuer 2010), “weight-based teasing/bullying” (Neumark-Sztainer et al. 2002; Puhl, Luedicke, and Heuer 2010), and “weight discrimination” (Paul and Townsend 1995; Roehling 1999). Some of these terms are not wholly synonymous, and they can interact in multiple ways, potentially reinforcing one another. For example, weight discrimination refers to differential treatment based on someone’s weight, whereas weight stigma refers to the discrediting of people based on their weight.

## Spectrum of Approaches to Stigma

**Fig. 1:**
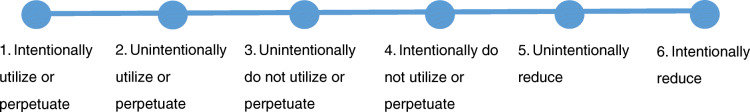
Spectrum of Approaches to Stigma

A range of approaches to stigma are possible and some of them are discernible within the academic literature on obesity. At one end of the spectrum are writers who encourage the use of weight stigma as a motivational tool to achieve weight loss and weight management across society. At the other end are writers who argue that stigmatization is not only ineffective as a weight loss tool but is actually harmful, and therefore weight stigma ought to be combatted directly (see Fig. 1).

The first position on the spectrum is to intentionally utilize or perpetuate stigma. Several writers argue for the active and intentional stigmatization of individuals perceived to be overweight or obese (e.g., Callahan 2013a; Freind 2012; Liddle 2013). The basic argument is as follows. Stigmatized individuals are marked as being outside the social norm. This leads those individuals to be treated poorly in various ways, which is unpleasant to experience, and this will motivate individuals to actively change to conform to the social norm (Callahan 2013a). Callahan seeks to replicate the “success” of anti-smoking campaigns, admitting that the “force of being shamed” and being “beat upon socially” to stop smoking were as persuasive to him as threats to his health (2013, 38). Callahan acknowledges that smoking is a behaviour, whereas weight and body size are not behaviours. Indeed, he notes that weight and body size are closely linked to character and selfhood (2013a, 38), and so to attack weight and body size is to attack people. However, he maintains that social pressure will push the public to accept the strong government interventions needed to change the ways they eat, exercise, and work so as to make inroads into obesity as a public health problem (39).^1^

The health promotion campaign Strong4Life featured black-and-white photographs of overweight children that resembled grim mug shots, with captions including “It’s hard to be a little girl if you’re not,” “Chubby isn’t cute. It leads to diabetes,” and “Big bones didn’t make me this way. Big meals did” (Fitbomb 2012). By portraying obesity as deeply discrediting and shameful, such materials utilize and perpetuate weight stigma. To use a different example, a campaign that focuses on personal responsibility for one’s weight can similarly perpetuate the stereotype that people with overweight or obesity lack discipline, tend to make unhealthy choices, and only have themselves to blame (Byrne and Niederdeppe 2012; MacLean et al. 2009; Saguy and Riley 2005). If this utilization and perpetuation of weight stigma is intentional, then the campaign occupies the first position on the spectrum; but if it is unintentional, then the campaign occupies the second position, which is to unintentionally utilize or perpetuate stigma. One is best placed to determine exactly which position a campaign adopts when intentions behind the campaign are documented and communicated.

The third position on the spectrum is to unintentionally not utilize or perpetuate stigma. This position must be included on the spectrum for conceptual completeness. For example, it is possible to imagine a health campaign that, simply by communicating accurate information about obesity, avoiding over-simplification and discrediting messaging, manages to not utilize or perpetuate weight stigma, even though weight stigma was never consciously considered when planning the campaign.

The fourth position on the spectrum is to intentionally not utilize or perpetuate stigma. To occupy this position is to consciously consider existing weight stigma and ensure that messaging does not exacerbate it. For example, exercise classes specifically designed for people with excess weight may be motivating, in that they may provide a sense of comradery for participants and lower the self-consciousness that may have previously acted as a barrier to physical activity. However, targeted intervention of this type may also imply that a particular group is in need of “fixing,” and this can be stigmatizing (“Oh, you go to *that* class”) (MacLean et al. 2009, 90). One can occupy the fourth position on the spectrum by keeping stigmatizing effects in focus or “on the table” (Saguy and Riley 2005) and by ensuring consistency and coherency in non-stigmatizing messages (MacLean et al. 2009, 92).

The fifth position on the spectrum is to unintentionally reduce stigma. For example, positive portrayals of individuals with overweight or obesity (individuals who demonstrate success, intelligence, or determination, say) can function to counter stigmatizing messages without being consciously aimed at achieving this.

The sixth position on the spectrum is to intentionally reduce stigma. To occupy this position is to consciously claim that weight stigma is harmful and therefore ought to be actively combatted and reduced. Academics increasingly occupy this position, as a growing body of evidence attests that weight stigma has deleterious effects on physical and mental health in a range of ways. According to Hatzenbuehler, “the accumulated literature makes a compelling case that stigma represents an additional burden that affects people above and beyond any impairments or deficits they may have” (Hatzenbuehler et al. 2013, 814).

## Matrix of Anti-Stigma Interventions

Corrigan et al. (2001) identified three intervention strategies to reduce stigma. First, protest strategies aim to “suppress negative representations and attitudes” through direct confrontation or explicit criticism (Corrigan et al. 2001, 187–188). Second, contact strategies facilitate constructive interactions between members of the public and members of the stigmatized group. Finally, education strategies aim to improve knowledge of stigmatized issues. We propose the addition of a fourth category, regulation strategies, to provide scope for legal and regulatory approaches, which typically seek to moderate discriminatory behaviour through justified coercion or, conversely, incentives.

Now recall the three mechanisms by which stigma operates: direct discrimination, structural discrimination, and psychological processes operating through the stigmatized person (self-stigma). Below we illustrate how considering these three generic mechanisms alongside the four anti-stigma intervention strategies just sketched serves to help map out different anti-stigma interventions. We are, in effect, multiplying or intersecting the three mechanisms with the four anti-stigma strategies to produce a novel output. What follows is a Matrix of Anti-Stigma Interventions, a conceptual framework aimed at improving the understanding, evaluation, and planning of interventions to reduce weight stigma and other forms of stigma (see Table 1).

**Table 1:** Matrix of Anti-Stigma Interventions

Generic Mechanism → Intervention Strategy ↓	Direct Discrimination	Structural Discrimination	Psychological Processes (Self-Stigma)
Protest	Condemn the discriminatory behaviour of an individual	Boycott an organization that has discriminatory policies or practices	Speak out against negative representations in the media
Contact	Facilitate contact with people in the stigmatized group who have obvious positive qualities	Increase the presence of stigmatized people in circles of power and influence	Participate in support groups (online or in person)
Education	Educate people about the harms of labelling and stereotyping and about how to not discriminate against others	Educate managers and people working with the public about the rights of individuals to be treated fairly and the legislation in place to protect those rights	Educate stigmatized people about the self-stigma process and teach them skills for building self-esteem and coping with discriminatory treatment
Regulation	Introduce anti-bullying and anti-discrimination policies that specify punitive measures for non-compliance	Introduce regulatory requirements or incentives for organizations to meet equal-opportunity targets	Empower media regulators to act against stigmatizing messages

The matrix builds on the works of Link (2001) and Corrigan et al. (2001), which pertain to stigma in general, not solely weight stigma, so the matrix is widely applicable. Each cell in the matrix contains an example of the use of one intervention strategy to counter one of the generic mechanisms of stigma. We acknowledge, however, that an intervention strategy may function to reduce stigma in multiple ways, especially in view of Link’s mechanisms being mutually reinforcing. The mutually reinforcing nature of Link’s mechanisms also suggests that an intervention or suite of interventions featuring multiple strategies—some combination of protest, contact, education, and regulation strategies—may prove most effective. This actually highlights the usefulness of the matrix, in that the matrix shows how different strategies can be used to target different mechanisms of stigma. In addition, the matrix can be used by a number of actors, including governments, non-government organizations, advocacy groups, and individuals, both to map out possible anti-stigma interventions in future and to better understand past or existing interventions.

An intervention adopting a particular strategy (protest, contact, education, or regulation) may have effects on more than one mechanism of stigma. For example, anti-bullying and anti-discrimination policies may have effects on direct discrimination as well as structural discrimination. However, if anti-stigma interventions commonly act on multiple mechanisms of stigma, then why is it helpful to present the full matrix, illustrating how each intervention strategy might target each stigma mechanism? Instead, could it not suffice simply to understand that there are three stigma mechanisms and interventions commonly have effects for two or all three of them? Such a general understanding risks glossing over possibilities in public health practice. Each cell in the matrix represents a distinctive possibility for a particular intervention strategy to act on a particular stigma mechanism. To stop short of systematically unpacking these possibilities risks missed opportunities for planning, understanding, and evaluating interventions. The anti-bullying and anti-discrimination policies that have effects on both direct and structural discrimination (and downstream even self-stigma) can nonetheless feature distinctive *components* that focus on a particular stigma mechanism, and appreciating this by way of the matrix can make for a more nuanced understanding of the policies. But perhaps more importantly, there will be policies and interventions whose planning or evaluation will problematically neglect one or more cells in the matrix. In particular, the matrix has the potential to alert intervention planners to possibilities that they might otherwise have glossed over. For example, in formulating anti-stigma regulation, a planner using the matrix can remember to at least consider a concerted regulatory approach to self-stigma, namely a policy component knowingly targeted at this and not simply at direct and structural discrimination. Or, to use a different example, a planner armed with the matrix might quickly glimpse the possibility of adding to their contact strategy an intervention component that targets self-stigma alongside direct discrimination, say by adding the simple but meaningful step of directing participants to support groups. Without the matrix, the planner might have neglected this and remained solely focused on direct discrimination when other possibilities abounded.

We now illustrate how the matrix can be used to structure thinking about anti-stigma interventions using the example of reducing weight stigma specifically. This involves unpacking individual cells in the matrix.

Using a protest strategy to reduce self-stigma, individuals and advocacy groups can speak out against misrepresentation, negative stereotyping, and stigmatizing messaging in the media. They can do this by directly contacting the offending media companies or by using social media to raise awareness of how stigmatizing messaging is problematic. For instance, on 6 April 2018, a British academic sent an open letter to the National Union of Journalists with the express support of many political, university, and advocacy actors (All-Party Parliament Group on Obesity, 2018). The letter highlighted stigmatizing media portrayals and argued that these conflicted with the Union’s Code of Conduct, which expresses a commitment not to incite hatred or discrimination and to ensure that information is accurate and fair (Flint 2018).

Several online blogs and social media groups^2^ function to provide a forum for people to discursively resist the stigmatizing messaging that they encounter in their daily life. This includes the discriminatory treatment that people experience, since discriminatory treatment itself sends a stigmatizing message. The online blogs also enact a contact strategy to reduce self-stigma by allowing participants to share their experiences and support one another.

Given the prevalence of obesity, it is likely that many individuals have some contact with individuals who have overweight or obesity, whether through interpersonal relationships or just in navigating the outside world. However, it is not necessarily the case that such contact promotes acceptance and tolerance, and therefore the nature of contact is significant: having positive contact with people who have overweight or obesity and are successful, intelligent, charismatic, and so on may help to counter the stereotyping that contributes to mistreatment. This equates to using a contact strategy to reduce direct discrimination.

Using an education strategy to reduce direct discrimination, schools and workplaces can provide training and education about fair treatment and the importance of not discriminating against others. MacLean et al. (2009, 91) note the importance of educating and training professionals, such as doctors, nurses, and educators, about stereotyping. However, it is well documented that educational approaches to reducing weight stigma have been resoundingly ineffective (Bell and Morgan 2000; Musher-Eizenman et al. 2004; Sigelman 1991; Sigelman, Miller, and Whitworth 1986). This may be, in part, because past educational interventions were not administered for long enough or without adjoining intervention modes that might permit the development of empathy, for example role-play or contact with stigmatized people.

Using an education strategy to reduce structural discrimination could take the form of educating managers, teachers, and healthcare providers about the rights of individuals to be treated fairly and the legislation in place to protect those rights. Clearly this requires that such legislation be in place, and this legislation equates to the use of a regulation strategy to reduce direct discrimination and probably structural discrimination as well.

Using an education strategy to reduce self-stigma could take the form of explaining relevant psychological processes to stigmatized people and highlighting that negative messages, such as those present in the media, can influence how we perceive ourselves, reducing our sense of self-worth. It could also take the form of promoting self-esteem building in general. The availability of counselling in schools and workplaces could further help individuals to develop coping skills when faced with weight-related teasing or discrimination. If healthcare providers were made more aware of self-stigma in patients, then they could potentially better monitor for self-esteem issues and body dissatisfaction, then recommend counselling or other measures as needed.

Using a regulation strategy to reduce direct discrimination takes the form of implementing and enforcing policies to moderate behaviour and reduce the incidence of discriminatory treatment. This is commonplace in schools and workplaces, for example. Using a regulation strategy to reduce structural discrimination takes the form of legislation such as equal opportunity acts, which are in place in relation to ethnicity and disability (for example, Australia’s *Racial Discrimination Act 1975* and *Disability Discrimination Act 1992* and South Australia’s *Equal Opportunity Act 1984*). Legislation can protect stigmatized groups from systemically discriminatory hiring practices, by way of further example.

Using a regulation strategy to reduce self-stigma can take the form of empowering regulatory bodies to take proportionate punitive action against media companies that broadcast stigmatizing messages. For example, any concerns or complaints regarding news, programmes, or advertisements shown on Australian television can be directed to the Australian Communications and Media Authority. Any punishment and consequent reduction of stigmatizing messages would result in fewer sources being available to reinforce and perpetuate self-stigma. Social media providers can also self-regulate by implementing and enforcing policies aimed at suppressing or countering stigmatizing messages. Ethical and political complexities concerning censorship need to be navigated in both cases.

In practice, someone evaluating or planning an anti-stigma intervention can look at the matrix and identify the cells that most apply. Which columns are most relevant or important given the focus of the intervention? Now, which rows? Once the key cells have been identified, the contents of those cells can then be evaluated or worked out in terms of the planned anti-stigma intervention. In this way, the matrix provides a structure for anti-stigma intervention evaluation and planning that may facilitate more systematic progress. In particular, it guides people to target known stigma mechanisms, reminding people of a number of potential mechanisms. Likewise, the matrix guides people to mobilize known intervention strategies, again reminding people that more than one is available. In this way, the matrix can also be used to identify gaps and opportunities when it comes to reducing stigma. If one or more stigma mechanisms (columns) are not being targeted, then this represents a missed opportunity or gap that might be closed via future intervention. Meanwhile, if one or more intervention strategies (rows) are not being mobilized, then this represents an opportunity to mobilize different or more diverse strategies, which may increase the overall effectiveness of anti-stigma efforts.

## Understanding and Building on Past Interventions to Reduce Weight Stigma

We now demonstrate how the matrix can be used to understand past interventions, say for the purpose of conducting an evaluation. Some programmatic attempts to reduce weight stigma have been made, with mixed results. We examine selected examples that allow us to most clearly demonstrate the usefulness of the matrix in evaluating these. In this section, we also draw out the most promising intervention elements that may benefit future interventions aimed at reducing weight stigma.

Simplistic beliefs about obesity aetiology contribute to weight stigma, especially the beliefs that obesity results from laziness, gluttony, and a lack of self-discipline and that accordingly overweight individuals should be held responsible for their weight (Bell and Morgan 2000; Dejong 1980, 1993; Musher-Eizenman et al. 2004). To counter simplistic beliefs, interventions that provide accurate information about obesity have been implemented, especially amongst young children.

Very Important Kids was an intervention designed to reduce teasing and weight stigma among children in grades four, five, and six. It incorporated an after-school programme and theatre production for students, staff training, a no-teasing campaign, and various levels of family involvement. Referring to the matrix, the intervention used an education strategy targeted at direct discrimination. While the intervention saw positive results in the reduction of overall teasing, the reduction of weight-based teasing specifically was minimal (Haines, Neumark-Sztainer, Perry, et al. 2006b). The successes of the intervention may have been due to so many students participating and the messages being sustained and consistent (Haines, Neumark-Sztainer, Perry, et al. 2006b, 890). This lesson should be remembered when planning similar anti-stigma interventions.

Eating Disorders Awareness and Prevention (EDAP) developed a puppet programme for children aged six to ten years to promote acceptance of a diverse range of body shapes, healthy attitudes about food and eating, and a healthy self-concept (Irving 2000). The programme used “scripts” to address issues that contribute to disordered eating, including emotional distress, body acceptance, and dieting (Irving 2000, 223). Referring to the matrix, the intervention used an education strategy targeted at direct discrimination and psychological processes (self-stigma). The EDAP puppet programme showed promising results. Student evaluations indicated that the programme successfully discouraged teasing in all forms, not just related to body shape and size. The programme also successfully encouraged students to treat everybody well, including themselves. Negative attitudes towards larger bodies were reduced, as larger bodies were evaluated more favourably post-programme. It is possible that the programme’s creative engagement with students contributed to its success. Again, this lesson should be remembered when planning similar anti-stigma interventions.

Familiarity with the matrix here alerts one to the absence of other intervention strategies and targeted mechanisms. For instance, perhaps the interventions could have readily added compatible strategies, such as a contact strategy targeted at direct discrimination via a person with obesity interacting positively with participants. The interventions also neglected to target structural discrimination: adults leading the school and community could have been educated about the rights of people with obesity, for example. This analysis shows how the matrix can be used to identify gaps and opportunities when it comes to reducing stigma.

## Conclusion

Weight stigma can be approached in a range of ways, from intentionally utilizing or perpetuating weight stigma in attempts to reduce obesity to intentionally reducing weight stigma, partly to achieve the same end. By placing these approaches along a spectrum (see Fig. 1), we have provided a conceptual framework for understanding the range of possible approaches to dealing with stigma in general.

The spectrum should not be misconstrued as implying that each position on it is equally valid or defensible, since this is not necessarily so. Evidence shows that some positions are neither valid nor defensible in the case of weight stigma. Weight stigma manifests in peer rejection and social isolation (Brewis 2014), teasing and bullying (Neumark-Sztainer et al. 2002), and the loss of opportunities across many domains such as education, employment, and health care (Bertakis and Azari 2005; Puhl and Heuer 2010; Roehling 1999; Spahlholz et al. 2016). Extensive empirical evidence has consistently demonstrated the harmful effects of weight stigma (Brewis 2014; Link 2001; Major and O’Brien 2005). Experiencing weight stigma contributes to poor health in a range of ways, including in the development of disordered eating and in acting as a barrier to physical activity and healthcare access (Hatzenbuehler et al. 2013; Nolan and Eshleman 2016; Puhl and Suh 2015). Weight stigma perpetuates weight gain and retention (Brewis 2014). Given these effects, the strategic use of weight stigma to try to motivate weight loss and reduce obesity (position one on the spectrum) is not merely ineffective but counterproductive. Only efforts to intentionally reduce weight stigma (position six on the spectrum) fully reckon with the empirical evidence.

Finally, we built on work by Corrigan et al. (2001) and Link (2001) to develop the Matrix of Anti-Stigma Interventions (see Table 1). This is a conceptual framework to help structure thinking about anti-stigma interventions in the case of weight stigma and beyond. The matrix provides anti-stigma intervention evaluation and planning with structure so as to more systematically make progress and achieve social change to improve public health. It guides people to target known stigma mechanisms, reminding people that there is more than one mechanism. Likewise, it guides people to mobilize known intervention strategies, again reminding people that there is more than one strategy. In this way, the matrix can be used to identify gaps and opportunities when it comes to reducing stigma.

## Footnotes

1 In response to criticism, Callahan (2013b) explained that he had made an “error in editing the manuscript” and that his main point was “to use social pressure on those not yet obese or just a little overweight to induce them to stay that way; that is, deploy it as a prevention strategy.” Callahan (2013b) stated that he does not “favor stigmatizing the overweight or obese, and surely not discriminating against them”. However, Voigt, Nicholls, and Williams (2014, 104) find fault, observing that Callahan had “restated his assumption” that a lot of weight-loss behaviour had been “spurred by stigma.”

2 For example, Fit is a Feminist Issue: Feminist Reflections on Fitness, Sport, and Health (https://fitisafeministissue.com/), Fit Fatties (https://www.facebook.com/groups/fitfatties/), Fierce Freethinking Fatties (https://fiercefatties.wordpress.com), Fat Heffalump: Living with Fattitude (https://fatheffalump.wordpress.com/), The Association for Size Diversity and Health—Health at Every Size Blog (https://healthateverysizeblog.org/).
